# Metabolic Changes and Their Associations with Selected Nutrients Intake in the Group of Workers Exposed to Arsenic

**DOI:** 10.3390/metabo13010070

**Published:** 2023-01-01

**Authors:** Monika Sijko, Beata Janasik, Wojciech Wąsowicz, Lucyna Kozłowska

**Affiliations:** 1 Laboratory of Human Metabolism Research, Department of Dietetics, Warsaw University of Life Sciences, 02776 Warsaw, Poland; 2 Department of Environmental and Biological Monitoring, Nofer Institute of Occupational Medicine, 91348 Lodz, Poland

**Keywords:** inorganic arsenic, metabolic pathways, diet, untargeted metabolomic

## Abstract

Arsenic (As) exposure causes numerous adverse health effects, which can be reduced by the nutrients involved in the metabolism of iAs (inorganic As). This study was carried out on two groups of copper-smelting workers: WN, workers with a urinary total arsenic (tAs) concentration within the norm (*n* = 75), and WH, workers with a urinary tAs concentration above the norm (*n* = 41). This study aimed to analyze the association between the intake level of the nutrients involved in iAs metabolism and the signal intensity of the metabolites that were affected by iAs exposure. An untargeted metabolomics analysis was carried out on urine samples using liquid chromatography–mass spectrometry, and the intake of the nutrients was analyzed based on 3-day dietary records. Compared with the WN group, five pathways (the metabolism of amino acids, carbohydrates, glycans, vitamins, and nucleotides) with twenty-five putatively annotated metabolites were found to be increased in the WH group. In the WN group, the intake of nutrients (methionine; vitamins B2, B6, and B12; folate; and zinc) was negatively associated with six metabolites (cytosine, D-glucuronic acid, N-acetyl-D-glucosamine, pyroglutamic acid, uridine, and urocanic acid), whereas in the WH group, it was associated with five metabolites (D-glucuronic acid, L-glutamic acid, N-acetyl-D-glucosamine, N-acetylneuraminic acid, and uridine). Furthermore, in the WH group, positive associations between methionine, folate, and zinc intake and the signal intensity of succinic acid and 3-mercaptolactic acid were observed. These results highlight the need to educate the participants about the intake level of the nutrients involved in iAs metabolism and may contribute to further considerations with respect to the formulation of dietary recommendations for people exposed to iAs.

## 1. Introduction

There are two major sources of As (arsenic) exposure: environmental and occupational. Occupationally exposed people are also subject to environmental exposure as they are also part of the general population (whose exposure occurs through environmental pollution, anthropogenic sources, and diet). As is used in numerous industries; hence, many professional groups are exposed to As, including steelworkers, miners, farmers, gardeners, workers involved in the production of glass or ammunition, and e-waste-recycling workers. Inhalation and dermal contact are the primary routes of occupational exposure [1,2,3]. During the mining and smelting of ore, dusts containing As are emitted. Increased concentrations of total As (tAs) in the urine were observed among copper-smelting workers [4,5].

As occurs in organic and iAs (inorganic As) forms, of which the latter is the more toxic form; hence, it is the primary focus of research [6,7]. The harmful effects of iAs on the human body are well documented and are associated with various diseases, including diabetes and heart, kidney, and neurodegenerative diseases, and may cause neurodevelopmental problems in children [8,9,10,11,12]. The latest research also pointed to the possibility of the accumulation of tAs in semen and blood serum in a group of men working in highly polluted areas, which is related to a decline in semen quality and, consequently, to male reproductive disorders [13]. Furthermore, iAs exposure is associated with lung, skin, bladder, and other cancers, and the carcinogenicity of iAs has been confirmed by the International Agency for Research Cancer (IARC) [14].

Many studies have investigated the influence of iAs on the human body with the aim of identifying methods for reducing its negative effects. One such approach is dietary modification, an area that is constantly being researched. Particular attention has been paid to the dietary compounds of methyl donors (methionine, betaine, choline, and folic acid) and cofactors of reactions (vitamins B2, B6, B12, and zinc), which are involved in carbon metabolism (OCM). S-adenosyl-methionine is synthesized via OCM - is used as a methyl group donor in iAs metabolism. In this process, iAs is converted to MMA (monomethylarsonic acid) and DMA (dimethylarsinic acid). iAs, MMA, and DMA may be the end products of these process and excreted in urine. The proportions of these metabolites reflect the efficiency of iAs metabolism. DMA is rapidly excreted in the urine compared to iAs and MMA; as such, its higher concentration in urine indicates more efficient metabolism [15,16]. Low iAs metabolism efficiency (higher urinary MMA concentrations) is associated with an increased risk of As-associated carotid atherosclerosis, urothelial carcinoma, and skin lesions, among other ailments [17,18,19]. Many studies, both on animal models and human populations exposed to iAs primarily via drinking water, have focused on the relationship between the intake of nutrients and the concentration of As metabolites in the urine. Some studies have shown that these nutrients can improve the efficiency of iAs metabolism (observed as decreased concentrations of iAs and MMA and an increased urinary DMA concentration) and reduce adverse health effects, but not all studies have provided conclusive results [20,21].

In addition, to the best of our knowledge, studies analyzing the association between nutrient intake and the severity of metabolic changes in copper-smelting workers are not available in the literature. Therefore, this study aimed to analyze the association between the intake level of the nutrients involved in iAs metabolism and the signal intensity of the metabolites in this group of workers.

## 2. Materials and Methods

### 2.1. Study Participants and Design

**Figure 1:**
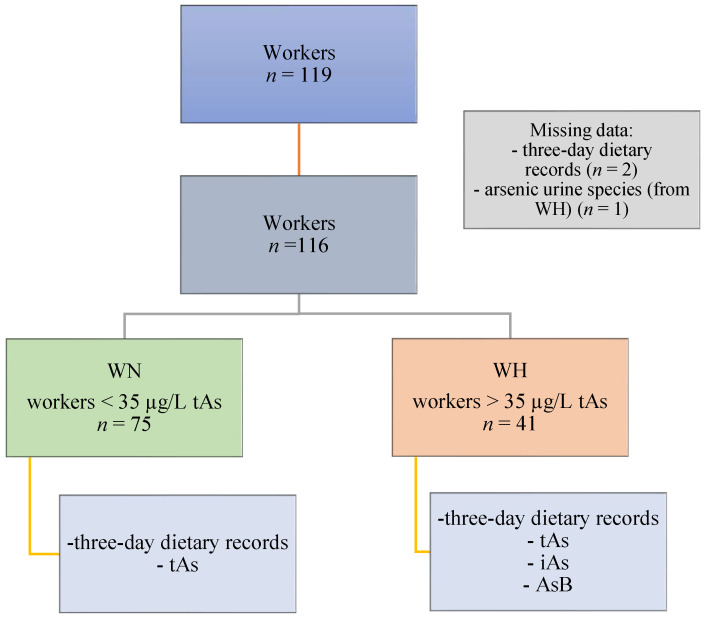
Study design. Abbreviations: AsB—arsenobetaine; iAs—inorganic arsenic; tAs—total arsenic; WN—the group of workers with urinary tAs concentration within the norm; WH—the group of workers with urinary tAs concentration above the norm.

This study was conducted on a group of copper-smelting workers from the southwestern region of Poland. The following inclusion criteria were enforced: occupationally exposed to iAs, male, age above 18, and presence at work during the study. Eligible workers from whom the following data were obtained were included in the analysis: 3-day dietary records of food and beverages consumed, questionnaire (with data on the subject’s age, height, body mass, and general characteristics), and urine samples. A total of 116 workers were included (3 participants were excluded from the analysis—2 of which were excluded due to missing information on dietary intake and 1 due to the lack of data on As speciation). Inhalation and dermal contact were determined to be the primary routes of occupational exposure. Then, the participants were divided into two groups (WN and WH) based on the concentration of tAs, according to the recommended Biological Limit Values for occupational exposure of 35 µg/L of urine [22]. The WN group consisted of workers with tAs concentrations <35 µg/L (*n* = 75), whereas the WH group consisted of workers with tAs concentrations >35 µg/L (*n* = 41). The study design is presented in Figure 1. All participants gave their informed consent for the study. Ethical approval was obtained from the Ethics Committee of the Nofer Institute of Occupational Medicine in Lodz, Poland (NR 08/2020).

### 2.2. Urine Collection and As Analysis

Urine samples were collected from workers after a shift at work. The samples were collected from May 2021 to June 2021 and were stored at −80 °C until As determination and untargeted analysis. In all urine samples, the concentrations of tAs and creatinine were determined. The concentrations of urinary tAs and As species were normalized to creatinine. In the WH group, speciation studies of two As forms—iAs and arsenobetaine (AsB)—were additionally performed. The concentration of urinary tAs was determined using an ELAN DRC-e ICP-MS with a Dynamic Reaction Cell (Perkin Elmer, SCIEX, Waltham, MA, USA), and the concentration of As species—iAs and AsB—were determined using the instrument Series 200 HPLC (Perkin Elmer, SCIEX, Waltham, MA, USA). Further description of As determination in the urine was provided in the study by Janasik et al. [23].

### 2.3. Diet Assessment

Dietary intake of the selected nutrients was analyzed based on 3-day dietary records of food and beverages consumed. The participants were instructed on how to fill in the dietary record (they received a standard template, which included time and place of consumption, name of the meal, ingredients, amount, or home measures) and were asked to accurately record all food products and beverages and report food’s preparation and portion sizes (using kitchen scales or typical household measures). For quantitative analysis, data from 3-day dietary records of consumed food and beverages were entered into the Dieta 6.0 (Warsaw, Poland) computer program, as recommended by the National Centre of Nutritional Education. In accordance with the international methodology of dietary research, the portion size was determined based on photographs of products and dishes included in the “Album of photos of products and dishes” [24]. Mean daily values were calculated from the data obtained from 3-day dietary records. The intake level of the nutrients involved in As metabolism, calculated using the Dieta 6.0 program, was compared with the nutrition standards for the Polish population using EAR [25]. The intake of nutrients was calculated per kg of body mass.

### 2.4. Sample Preparations for Untargeted Metabolomics

Urine samples from the WH and WN groups were randomized and divided into two batches. Samples for metabolomics analysis were prepared using the latest protocol [26]. Two assays were used: one for the extraction of nonpolar and semipolar metabolites (assay 1) and the other for the extraction of polar metabolites (assay 2). The procedure for assay 1 was as follows: 300 µL solvent (ice-cold water and methanol at a 50:50 ratio with internal standards benzoyl-D5 and L-phenylalanine 3,3-D2) was added to 100 µL of urine (thawed at 4°C), vortexed for 2 min, and centrifuged at 20,879 rpm for 20 min at 4 °C. The same steps were followed for assay 2, except that a different solvent was used, acetonitrile and methanol (50:50), with the same internal standards. After centrifugation, the supernatants (200 µL) were aliquoted into vials. Quality control (QC) samples were prepared from all samples, and equal amounts of aliquots (100 µL) of each urine sample were mixed; this mixture was used to monitor system stability (injected every 10 experimental samples). Each batch comprised the following: samples for the equilibration system (10), analyzed samples (59, 58), QC samples (8), and blanks (2). More detailed information on sample preparation and analysis is available in our previous study [27].

### 2.5. Metabolomics Analysis

The equipment used in the study comprised a Waters ACQUITY™ Ultra Performance LC system (Waters Corp., Milford, MA, USA) connected to a Synapt G2Si Q-TOF mass spectrometer (Waters MS Technologies, Manchester, UK) with an electrospray (ESI) source (Waters, Manchester, UK). Analyses were performed in positive and negative ionization modes on two columns: an ACQUITY UPLC HSS T3 and an ACQUITY UPLC BEH Amide. The chemical reagent, metabolomics analysis parameters, and the fast data-dependent acquisition method were used as described in a study by Kozłowska et al. [27]. Compared with this study, only the gradient in the ACQUITY UPLC BEH Amide column for ESI was changed in the present study with respect to ESI+: t = 0.0–2.0 min, 1% B; t = 2.0–3.0 min, from 1% to 12% B; t = 3.0–6.0 min, from 12% to 50% B; t = 6.0–8.0 min, from 50% to 95% B; t = 8.0–8.5 min, from 95% to 99% B; t = 8.5–10.5 min, 99% B; t = 10.5–11 min, from 99% to 1% B; t = 11.0–14.0 min, 1% B. Gradient for ESI-: t = 0.0–2.0 min, 1% B; t = 2.0–3.2 min, from 1% to 6% B; t = 3.2–5.0 min, from 6% to 60% B; t = 5.0–5.5 min, from 60% to 95% B; t = 5.5–6.0 min, from 95% to 99% B; t = 6.0–9.5 min, 99% B; t = 9.5–10.5 min, from 99% to 1% B; t = 10.5–13.0 min, 1% B.

### 2.6. Bioinformatics and Statistical Analysis

For feature detection, retention time correction, alignment, and putative annotation of compound classes, files were loaded into the Progenesis software. The default parameters set for UPLC-High Res (Waters, Milford, MA, USA) were used. Then, the dataset was filtered; accordingly, metabolite features with a blank contribution >5%, QC relative standard deviation >25%, and QC count and sample count <60% were removed. Unwanted variations (signal drift and batch effects) were nullified by normalization performed on the web platform MetaboGroupS (https://www.omicsolution.com/wukong/MetaboGroupS/ accessed on 11 May 2022) [28]. Among the seven methods of normalization using the k-nearest neighbor algorithm (which imputes missing values) and log2 transformation (which removes data skewness), the most effective was the Eigen MS method.

Significant pathways were analyzed using the Functional Analysis module in MetaboAnalyst 5.0 platform (https://www.metaboanalyst.ca/home.xhtml accessed on 20 June 2022) [29]. A peak list with the retention time and *p* values was used, and the following parameters were used in each mode: mass tolerance 5 ppm, mummichog algorithms, adducts for positive and negative modes, and pathways/metabolite sets containing at least two entries. Compounds belonging to significantly changed pathways were fragmented. For putative annotation of compounds (level 2), the obtained fragmentation spectra of the compounds were compared with the spectra available in the Human Metabolome Database [30].

Statistica software, version 13.0 (StatSoft Inc., Tulsa, OK, USA), was used for statistical analyses. Normality of distribution was assessed using the Shapiro–Wilk test and expressed as means ± standard deviations for parametric distribution or medians and min–max for nonparametric distribution. Student’s *t*-test for parametric distributions and Mann–Whitney U test for nonparametric distributions were used to compare variables between two groups. The Pearson correlation coefficient and the Spearman rank correlation coefficient were used to analyze the correlation between the intake of the nutrients involved in As metabolism and putatively annotated metabolites. Differences at *p* < 0.05 were considered significant.

## 3. Results

### 3.1. General Characteristic of the Workers

The overall characteristics of the participants are presented in Table 1. In brief, all the study participants (116) were male and worked in a copper-smelting plant. The participants were divided into two groups: WN, comprising workers with a urinary tAs concentration below 35 µg/L (*n* = 75), and WH, comprising workers with a urinary tAs concentration above 35 µg/L (*n* = 41). Between these groups, there were no significant differences in age, height, and period of iAs exposure; however, significant differences were observed concerning body mass and body mass index, with higher values in the WH group.

The concentrations of tAs and its species in the urine are presented in Table 2. Significant differences were observed in the urinary tAs between the WN and WH groups. The same results were obtained while using creatinine-adjusted urinary tAs concentrations. The WH group showed significantly higher concentrations of urinary tAs, both when adjusted and not for urinary creatinine, in which *p* = 0.0000 in both cases. The concentration of iAs in the urine was determined only in the WH group, which was almost twofold higher than the determined urinary concentration of AsB. When adjusted for urinary creatinine, the urinary concentration of iAs was nearly 2.5 times higher than that of AsB.

The daily intake of selected nutrients (mg/kg bm or µg/kg bm) involved in iAs metabolism, such as methionine; vitamins B2, B6, and B12; folate; and zinc, did not differ between the two groups (Table 3). Only the mean dietary folate intake was lower than the estimated average requirement (EAR). The intake of the remaining analyzed nutrients was above the EAR norms. The average daily intake of nutrients as per the EAR norms is presented in the Supplementary Materials (Table S1).

### 3.2. Differences in Metabolic Profile beetwen WN and WH

Compared with the WN group, a higher signal intensity was observed for the metabolites belonging to the five pathways in the WH group: amino acid metabolism, carbohydrate metabolism, glycan biosynthesis and metabolism, vitamin metabolism, and nucleotide metabolism (Table 4).

In the amino acid metabolism pathway, significant changes were noted in the following sub-pathways: aspartate and asparagine metabolism (*p* = 0.0168), histidine metabolism (*p* = 0.0168), and methionine and cysteine metabolism (*p* = 0.0176). In the carbohydrate metabolism pathway, the following sub-pathways showed significant changes: butanoate metabolism (*p* = 0.0135), glycolysis and gluconeogenesis (*p* = 0.0039), pentose and glucuronate interconversions (*p* = 0.0427), and propanoate metabolism (*p* = 0.0463/0.0003). The following significant changes were noted in the sub-pathways of the following pathways, in which the sub-pathways are presented after each pathway: in the glycan biosynthesis and metabolism pathway—heparan sulfate degradation (*p* = 0.0082), keratan sulfate degradation (*p* = 0.0313), and hyaluronan metabolism (*p* = 0.0063); in the metabolism of vitamin pathways—vitamin B2 metabolism (*p* = 0.0399), vitamin B6 metabolism (*p* = 0.0496), and vitamin B9 metabolism (*p* = 0.0047); and in the nucleotide metabolism pathway—pyrimidine metabolism (*p* = 0.0195 in negative mode; 0.0312 in positive mode).

### 3.3. Relationship between Dietary Nutrients Intake Involved in iAs Metabolism and Signal Intensity of Putatively Anotated Metabolites 

The associations between the intake of dietary nutrients involved in iAs metabolism and the putatively annotated metabolites belonging to significantly changed pathways in the analyses of the differences between WN and WH groups were analyzed. A correlation analysis was conducted on the whole group as well as separately in the WH and WN groups. Several dependencies between the analyzed nutrients and 11 metabolites were observed (Table 5).

Overall, in both groups, the intake of all the analyzed nutrients (methionine, vitamin B2, B6 and B12, folate, and zinc) was negatively associated with the following eight metabolites: cytosine, D-glucuronic acid, hydroxypropionic acid, N-acetyl-D-glucosamine, N-acetylneuraminic acid, pyroglutamic acid, uridine, and urocanic acid.

In the WN group, negative correlations were observed between the intake of all the analyzed nutrients and the following six metabolites: cytosine, D-glucuronic acid, N-acetyl-D-glucosamine, pyroglutamic acid, uridine, and urocanic acid.

In the WH group, the intake of nearly all the analyzed nutrients (except zinc) was negatively correlated with the following five metabolites: D-glucuronic acid, L-glutamic acid, N-acetyl-D-glucosamine, N-acetylneuraminic acid, and uridine. Moreover, in this group, positive correlations were observed between methionine, folate, and zinc intake, and the signal intensity of succinic acid and 3-mercaptolactic acid.

In both the WN and WH groups, the intake of the analyzed nutrients was negatively associated with the signal intensity of the following three metabolites: D-glucuronic acid, N-acetyl-D-glucosamine, and uridine. In contrast, the intake of the nutrients was negatively associated with the signal intensity of the following three metabolites: cytosine, pyroglutamic acid, and urocanic acid in the WN group, with no significant relationships in the WH group. However, negative correlations were observed in the WH group between the intake of the analyzed nutrients and the signal intensity of two metabolites, namely, L-glutamic acid and N-acetylneuraminic acid, which were not statistically significant in the WN group.

## 4. Discussion

To the best of our knowledge, this is the first metabolomics study on copper-smelting workers. Moreover, this is also the first study in which the association between the intake of the nutrients involved in iAs metabolism and changes in metabolic profile has been analyzed. Significant changes in metabolism were observed in the group of workers exposed to iAs. A total of 25 putatively annotated metabolites belonging to significantly changed pathways were detected in the analysis of differences between the WN and WH groups. In addition, associations between the intake of the nutrients involved in iAs metabolism and 11 putatively annotated metabolites were observed. To clearly understand the findings of this study, this article’s discussion was divided into two parts. First, alterations in the metabolism under exposure to iAs were discussed, and secondly, associations between the intake of the nutrients and putatively annotated metabolites were discussed.

### 4.1. Urinary Metabolomics

The findings of this study are consistent with those of previous urinary metabolomics studies [31,32,33], which showed that As exposure is associated with numerous changes in metabolism in adults. In the present study, a higher signal intensity was observed in the WH group with respect to several metabolites belonging to the amino acid, carbohydrate, glycan, vitamin, and nucleotide pathways compared with the WN group. These results seem to be consistent with those obtained by Zhang et al. [32], who reported changes in nucleotide (guanine) and amino acid (serine, hippurate, and acetyl-N-formyl-5-methoxy kynurenamine) metabolism. Wu et al. [33] also found alterations in amino acid metabolism (glycine, L-threonine, and serine) and identified changes in the signal intensity of succinic acid and pyroglutamic acid; these findings are consistent with the results of the present study. Kozłowska et al. [31] reported that the signal intensity of several metabolites is higher in men and women with higher urinary tAs concentrations and that these metabolites are also associated with amino acid, vitamin, and nucleotide metabolism. In these three metabolomics studies, changes were also observed in other pathways, which were dependent on the differences in the source and level of As exposure. In a study by Zhang et al. [32], the median tAs concentration was 40.03 µg/g of creatinine in men with environmental exposure. However, in a study by Wu et al. [33] on men and women exposed to As via drinking water (<50 µg/L), higher baseline tAs concentrations were observed (194.30 µg/g of creatinine in the male group and 206.70 µg/g of creatinine in the female group). Kozlowska et al. [31] conducted a study on a group of adults and children environmentally exposed to As and reported urinary tAs concentrations in a wide range, namely, 16.40–170.13 µg/g. Other factors such as the concentration of iAs in the urine, the duration of exposure to iAs, and the age and gender of the participants, as well as the use of different analytical techniques (HPLC–MS, UPLC–MS, and GC–MS), might have influenced the differences in the obtained results.

### 4.2. Association between Intake of Nutrients Involved in iAs Metabolism and Putatively Annotated Metabolites

The major finding of this study was the relationship between the intake level of the nutrients involved in iAs metabolism (methionine; vitamins B2, B6, and B12; folate; and zinc) and 11 putatively annotated metabolites belonging to significantly changed pathways in the analyses of differences between the WN and WH groups. Methionine; vitamins B2, B6, and B12; folate; and zinc are cofactors and donors of methyl groups in the metabolic changes of iAs to MMA and DMA. Many studies have reported correlations between the efficiency of iAs methylation and the spectrum of adverse changes associated with iAs exposure [21]. Thus, the relationships observed between dietary intake and the signal intensity of the metabolites may also indirectly reflect the efficiency of methylation and thus the severity of the adverse changes associated with iAs exposure. Due to the lack of findings regarding correlations between the intake of the aforementioned nutrients and changes in the metabolic profiles of the individuals exposed to iAs, this discussion was focused on analyzing the effects of increased concentrations of these metabolites and the benefits of increasing the intake of methyl group donors and cofactors of iAs metabolism.

The signal intensity of N-acetyl-D-glucosamine correlated negatively with the intake of methionine; vitamins B2, B6, and B12; and zinc. In vitro and in vivo studies have reported that As exposure increases the concentration of this metabolite. In a group of rats exposed to outdoor air pollution (containing, inter alia, As), a higher concentration of N-acetyl-D-glucosamine in the serum was also observed than in the control group. Moreover, this metabolite was positively related to the phosphorylation of H2AX at Ser 139 (γ-H2AX) in the lungs, which is one of the biomarkers of deoxyribonucleic acid (DNA) damage [34]. Interestingly, the oral administration of N-acetyl-D-glucosamine in mice increased DNA damage in the pancreas, brain, kidney, liver, lungs, and colon. In addition, in various nontumorigenic cell lines, treatment with N-acetyl-D-glucosamine for 3 days resulted in genome instability [35]. In a study by Ni et al. [36] on a group of workers exposed to iAs, DNA damage to the P21 gene fragments was observed, which was associated with the reduced methylation of iAs (positively associated with the percentage of MMA and negatively with the percentage of DMA in the urine). The results of the aforementioned study indicated that iAs exposure is associated with an increase in N-acetyl-D-glucosamine signal intensity. In accordance with this finding, based on the correlations observed in the present study between nutrient intake, N-acetyl-D-glucosamine, and DNA damage, it seems that a higher intake of these nutrients may be related to a reduction in the severity of these processes.

In this study, the intake of vitamins B2, B6, and B12 as well as zinc was negatively correlated with the signal intensity of pyroglutamic acid. This signal intensity was higher in the WH group than in the WN group. In metabolomics studies on populations exposed to various chemical compounds, differences in the signal intensity of this metabolite were observed between exposed groups and reference groups. A lower signal intensity of pyroglutamic acid in the urine was observed in Bangladeshi adults chronically exposed to As. Moreover, the signal intensity of this metabolite was inversely associated with the As concentration in the urine and drinking water [33]. In addition, in a urinary metabolomics study by Zeng et al. [37], pyroglutamic acid was downregulated in a group of women exposed to cadmium, which was negatively correlated with urinary cadmium concentrations. A serum metabolomics analysis also showed the downregulation of pyroglutamic acid in children and adolescents exposed to multiple carcinogens (including As). In addition, this metabolite was associated with biomarkers of early health effects (inversely with oxidative stress biomarkers, namely, 8-hydroxy-2′-deoxyguanosine and 4-hydroxy-2-nonenal-mercapturic acid, and inversely and positively with three acylcarnitines) [38]. The findings of the present study are consistent with those of a metabolomics study on women with higher cadmium concentrations, wherein a higher signal intensity of pyroglutamic acid was observed [39]. Similarly, in a study on rats exposed to iAs, an increased signal intensity of this metabolite was observed in the serum [40]. An in vitro study on rat brains showed that pyroglutamic acid may cause oxidative stress by reducing nonenzymatic antioxidant capacity, thus causing oxidative damage to proteins and increased reactive species in rat brain [41]. Another study reported oxidative stress in a group of workers exposed to As, which was related to decreased total and native thiol concentrations and an increased disulfide concentration in the serum [42]. The present findings suggest that As exposure is associated with a change in pyroglutamic acid signal intensity, which may also be related to the severity of oxidative stress. Based on these results and the negative correlations observed between the nutrient intake and the signal intensity of this metabolite, it seems that the higher intake may be related to a reduction in the severity of oxidative stress.

Negative relationships were observed between the intake of vitamin B2, vitamin B6, and folate and the signal intensity of L-glutamic acid. L-glutamic acid and pyroglutamic acid are related metabolites. Pyroglutamic acid can be produced by L-glutamic acid in the presence of enzymes, and the reaction can also proceed in the reverse direction, i.e., enzyme 5-oxoprolinase hydrolyzes pyroglutamic acid to yield L-glutamic acid [43]. In the present study, a higher L-glutamic acid signal intensity was observed in the WH group compared with the WN group, which is consistent with other studies. In murine models orally administered iAs, an increased signal intensity of L-glutamic acid was observed in the liver [44] as well as in the plasma [45]. In addition, in men and women exposed to cadmium, L-glutamic acid in the urine was upregulated and correlated with an increase in cadmium concentrations [37]. In a targeted metabolomics study in which dependencies between As metabolism and diabetes were analyzed, positive associations between the homeostasis model assessment index (HOMA2-IR), waist circumference, and the plasma level of L-glutamic acid were observed [46]. The relationship between diabetes and L-glutamic acid was confirmed in several studies [47,48,49]. Furthermore, two meta-analyses showed the association between iAs exposure and increased diabetes mellitus risk [8,50]. However, in a study by Spratlen et al. [46], L-glutamic acid was associated with more efficient As metabolism (through increased %DMA, decreased %MMA, and %iAs in the urine). Based on these findings, exposure to iAs is related to an increased signal intensity of L-glutamic acid. Considering the correlations observed in our study and the findings of other studies regarding the association between L-glutamic acid and diabetes, it seems that a higher intake of vitamins B2 and B6 and folate may be an important modulator of the development of the aforementioned disorders.

This study showed positive associations between the intake of methionine, folate, and zinc and the signal intensity of succinic acid, in which the signal intensity of this metabolite was higher in the WH group than in the WN group. Another urinary metabolomics study performed on a group of adults exposed to As reported that the signal intensity of succinic acid was inversely associated with As concentrations in urine and water, with a higher urinary tAs concentration (194.3 µg/g of creatinine in men and 206.7 µg/g of creatinine in women), which may affect the differences in the obtained results [33]. However, in another untargeted metabolomics study on an As-exposed population, an increased signal intensity of succinic acid and argininosuccinic acid was observed in boys and men with higher tAs concentrations, respectively [31]. In studies on animal models, increased concentrations of this metabolite in the urine were observed in diabetic mice [51], as well as in the plasma in an animal model of hypertension and metabolic disease, but such increases were not observed in hypertensive nor in diabetic human participants [52]. Under normal conditions, succinic acid undergoes various reactions. Apart from being involved in butanoate metabolism, it is involved in the tricarboxylic acid cycle (TCA cycle). In the TCA cycle, acetyl coenzyme A is oxidized, which causes the release of energy in the form of adenosine triphosphate. On the other hand, succinic acid is converted into fumaric acid by the enzyme succinate dehydrogenase, and in case of a dysfunction of this step, succinic acid is accumulated and can act as an oncometabolite [53,54,55]. This metabolite may also contribute to inflammation (acting as immune signaling), which has been observed in mouse macrophages [56].

In the present study, a negative relationship was observed between the intake of vitamins B2, B6, and B12 and folate and the signal intensity of D-glucuronic acid. Disorders in the glucuronate degradation sub-pathway were also reported in another metabolomics study on a population exposed to As, in which a higher signal intensity of L-threo-2-pentulose belonging to this sub-pathway was observed in men, women, and boys with high As concentrations [31]. The signal intensity of D-glucuronic acid in the serum was associated with mortality in patients with cirrhosis [57]. Furthermore, in patients with diabetes mellitus, hepatocellular carcinoma, and liver cirrhosis, an increased level of glucuronic acid was observed in the serum [58,59]. In a study by Ho et al. [60], the level of glucuronic acid increased with age, and circulating glucuronic acid was considered to be a biomarker of biological aging and a predictor of all-cause mortality and health-related outcomes. Studies have shown that the microbiome can also affect the rapid cleavage of glucuronide conjugates by increasing the concentration of D-glucuronic acid and thus disrupting glucuronidation [60,61]. Considering the results of these studies, the higher signal intensity of D-glucuronic acid observed in the WH group in the present study may be associated with impaired glucuronidation and the risk of developing several diseases. The negative correlation of the consumption of cofactors of iAs metabolism and donors of methyl groups with this metabolite emphasizes the need for studies aimed at an in-depth understanding of these processes.

This study showed negative relationship between the intake of vitamins B2, B6, and B12 and the signal intensity of cytosine and uridine. The signal intensity of these metabolites was higher in the WH group than in the WN group. These findings are consistent with those of other studies, as shown by a study in which cytosine was upregulated in the urine of adults with high exposure to heavy metals (As, among others) and polycyclic aromatic hydrocarbons [62]. In men environmentally exposed to As, a positive correlation was reported between the concentration of As, male infertility, and uridine in the urine [63]. Cytosine, thymine, adenine, guanine, and uracil are the nitrogenous bases that build DNA and ribonucleic acid (RNA). Uracil, one of the nitrogenous bases of RNA, forms uridine when combined with ribose. A study by Zhang et al. [32] reported an increased signal intensity of one of the purine bases, guanine. These authors suggested that this change may indirectly indicate an increase in oxidative stress, which can lead to DNA damage. One marker of this damage is 8-oxoguanine, which is formed by the oxidation of guanine. Guanine combines by hydrogen bonding with cytosine, both of which are complementary nitrogenous bases, whereas 8-oxoguanine can combine with adenine, which leads to a point mutation [64]. Guanine-related disorders are an example of changes that can arise due to other nitrogenous bases that build DNA or RNA. Depending on the correlations between vitamin intake and the intensity of cytosine and uridine signaling observed in the present study, and the findings regarding guanine and DNA damage, it seems that a higher intake of these vitamins may have an indirect effect on reducing oxidative stress.

In the present study, negative relationships were observed between the intake of methionine and vitamin B12 and the signal intensity of hydroxypropionic acid. In metabolomics studies related to exposure to various compounds, no changes in the signal intensity of this metabolite were observed, but such changes have been reported in people with various types of cancer. A high urinary concentration of hydroxypropionic acid is a diagnostic biomarker of bladder and colorectal cancer [65,66]. In a study by Ikeda et al. [67], the signal intensity of hydroxypropionic acid in the serum was higher in gastric cancer patients compared with the control group. The IARC has classified iAs as carcinogens and indicated that iAs exposure can lead to lung, urinary bladder, and skin cancers [14]. In addition, studies have reported that long-term iAs exposure is associated with an increased risk of illness or increased mortality from gastric and colorectal cancers, especially in high-exposure regions [68,69]. Therefore, hydroxypropionic acid may be one of the potential biomarkers of cancers, whose risk of development is increased in people exposed to iAs. These findings, as well as the negative correlations between iAs intake and this metabolite observed in the present study, suggest the need for further research.

In the present study, a positive relationship between folate and zinc intake and the signal intensity of 3-mercaptolactic acid was observed. This metabolite is involved in the cysteine metabolism pathway, and its high signal intensity may suggest the intensification of changes in this pathway. Only a few studies have been conducted on this metabolite, but it seems that cysteine is involved in the metabolism of As, as suggested by the results of a study by García-Sevillano et al. [44]. In this study, using an animal model exposed to iAs, an increased signal intensity of cysteine was observed in the liver [44]. Spratlen et al. [46] reported a positive association of cysteine with the percentage of DMA and a negative association with the percentages of MMA and iAs, as well as with diabetes-related adverse outcomes. However, a regression analysis did not confirm these associations. These authors suggested that as cysteine is part of glutathione, its increased intensity may reflect the need for increased glutathione synthesis. Other studies showed that a higher cysteine intake is associated with increased urinary tAs excretion [70] and with lower percentages of urinary iAs and a higher quantity of first methylation steps (MMA:iAs) [71]. The positive relationship between folate and zinc intake and the signal intensity of 3-mercaptolactic acid, with an indirect relationship with cysteine, may suggest that their higher intake may be a modulator of iAs metabolism.

Negative relationships were observed between the intake of vitamins B2, B6, and B12 and the signal intensity of N-acetylneuraminic acid. In another study, both the signal intensity and concentration of N-acetylneuraminic acid were higher in the plasma of coronary artery disease patients, and the authors indicated this metabolite as a possible marker of this disease’s progression [72]. Lee et al. [73] conducted a meta-analysis revealing that urinary N-acetylneuraminic acid was associated with a higher risk of lung cancer; hence, these authors also indicated this metabolite as a potential biomarker of this disease. In addition, an increased concentration of N-acetylneuraminic acid in the urine has been observed in patients with diabetic nephropathy [74] and renal diseases [75]. These findings indicate that the upregulation of N-acetylneuraminic acid is related to coronary artery diseases, diabetic nephropathy, renal diseases, and lung cancer. These diseases can also develop as a result of exposure to iAs [8,9,14,76]. Given the results of these studies, in which a relationship between N-acetylneuraminic acid, the occurrence of the aforementioned disease, and iAs exposure was observed, it seems that vitamins B2, B6, and B12, being cofactors of many processes, can modulate the rate of many changes in their development.

In the present study, negative relationships were observed between the intake of methionine, vitamin B6, folate, and zinc and the signal intensity of urocanic acid. A higher signal intensity of this metabolite was observed in the WH group than in the WN group. In the metabolomics studies available in the literature, different results were reported: a lower signal intensity of urocanic acid in the urine was observed in psoriasis patients and atopic asthmatic children [77,78], whereas a higher signal intensity was observed in patients with endometrial carcinoma [79]. Urocanic acid is found in the skin, and it can be converted from the trans form to the cis form under the influence of ultraviolet radiation. It can have both beneficial and adverse effects on the body, for example, cis-urocanic acid can reduce cell-mediated immunity, leading to the development of skin cancer. However, due to urocanic acid’s acidifying properties towards the cytosol of cancer cells, it can be used to treat some cancers [80]. In studies on human participants, keratinocytes led to cis-induced reactive oxygen species generation, lipid oxidation, increased cytokine protein production, and the upregulation of genes associated with apoptosis, cell growth arrest, cytokine synthesis, and oxidative stress [81,82]. Exposure to iAs (through drinking water) increases the risk of skin lesions [83,84], and many studies have focused on a deeper understanding of the mechanisms of As-induced skin lesions and cancers [85,86]. The findings of studies on human keratinocytes, the relationship between iAs exposure and skin lesions, and the relationship between the intake of methionine, vitamin B6, folate, and zinc and the signal intensity of urocanic acid observed in the present study indicate the need for further research in this area.

**Figure 2:**
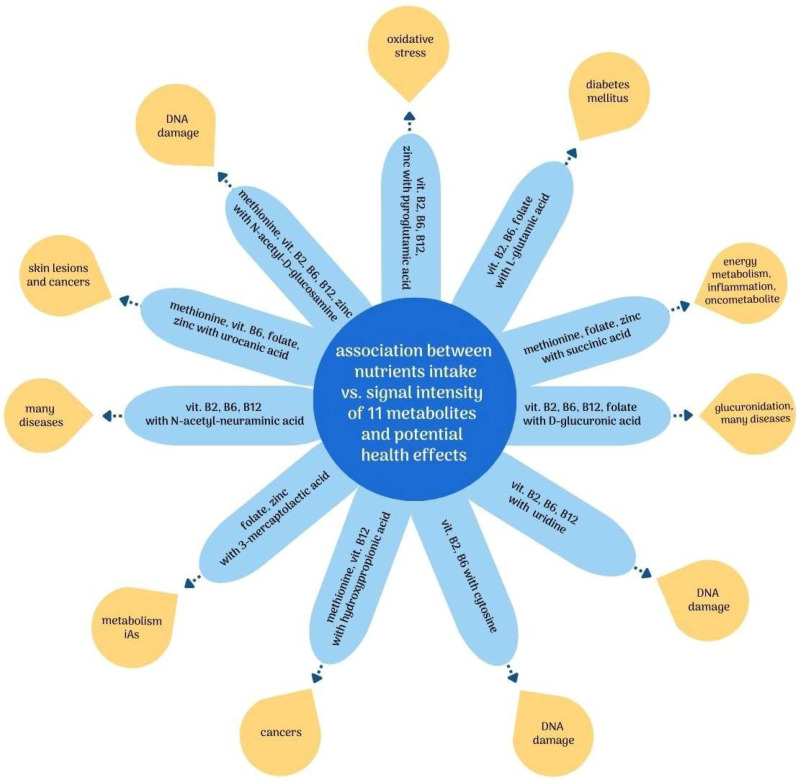
Association between intake of nutrients involved in iAs metabolism vs. putatively annotated metabolites and related potential negative health effects. Abbreviations: DNA—deoxyribonucleic acid; iAs—inorganic arsenic; vit.—vitamin.

Considering the observed negative correlations, it can be presumed that higher intakes of methionine; vitamins B2, B6, and B12; folate; and zinc, through their influence on the metabolism of iAs, may also have an indirectly beneficial effect by reducing the severity of the adverse effects of iAs exposure. Figure 2 summarizes the observed relationships between nutrient intake and potential adverse health effects associated with the putatively annotated metabolites.

### 4.3. Strengths and Limitations

The strengths and limitations of this study should be considered while interpreting its results, which are listed below.

Strengths:

- The determination of urinary tAs concentration using well-developed methods;
- The analysis of several nutrients involved in As metabolism;
- Comprehensive analysis of the metabolic profile of workers exposed to iAs (not only urinary As metabolites), which allows for a deeper understanding of the mechanisms that occur during exposure;
- First study to combine the amount of nutrient intake and metabolomics data, which may fill the research gap and provide a direction for further research.

Limitations:

- This study only included men; thus, the results cannot be generalized to the entire population;
- The analysis solely concerned exposure to iAs, without considering the exposure to other compounds that could have influenced the results;
- The disadvantages related to 3-day dietary records hampered the acquirement of certain findings, and include: an underestimation of intake; the failure to account for the seasonality of intake; possible differences between the 3-day records and typical consumption; the fact that the analysis of the consumption of nutrients was based solely on diet, not including dietary supplements (48.3% of the respondents declared their use); and the consumption of rice, seafood, and fish, which may have interfered with the results due to their high As content (however, only 22.4% declared consuming fish in the last 48 h);
- Only one urine sample was taken from each participant (no multiple measurements/serial exposure data);
- Urinary concentrations of DMA and MMA were not determined.

Some of these limitations might have led to the underestimation of the relationship between the intake of nutrients and the putatively annotated metabolites. This research is a starting point and can serve as a groundwork for future studies.

## 5. Conclusions

Between the two groups of copper-smelting workers, namely, WN and WH, differences in their metabolic profiles were observed. Compared with the WN group, five pathways (the metabolism of amino acids, carbohydrates, glycans, vitamins, and nucleotides) with twenty-five putatively annotated metabolites were found to be increased in the WH group. In both study groups, negative correlations were observed between the intake of methionine; vitamins B2, B6, and B12; folate; and zinc and the signal intensity of the putatively annotated metabolites. Considering these correlations, it seems that a higher nutrient intake may reduce the severity of the adverse processes and disorders associated with iAs exposure. The findings of the present study indicate the need to educate the participants about the intake level of nutrients involved in the metabolism of iAs. These findings may contribute to further considerations during the development of dietary recommendations for people exposed to iAs. If the results of these analyses are confirmed in further studies, changes in diet could be the basis for reducing the adverse effects of exposure to iAs.

## Figures and Tables

**Table 1:** General characteristics of the workers exposed to iAs.

Parameter *	Both Group	WN	WH	*p* Value **
	*n* = 116	*n* = 75	*n* = 41	
Age (years)	43.5 (21.0–62.0)	42.1 ± 10.0	44.0 (23.0–56.0)	0.4772
Height (cm)	177.0 (165.0–198.0)	176.5 (165.0–198.0)	178.3 ± 6.1	0.9673
Body mass (kg)	89.4 ± 14.8	86.7 ± 14.0	94.0 ± 15.1	0.0109
BMI (kg/m^2^)	28.1 ± 4.3	27.2 ± 4.1	29.5 ± 4.2	0.0047
Period of iAs exposure (years)	17.5 (1.0–44.0)	17.0 (1.0–44.0)	18.5 (2.0–38.0)	0.7867

Abbreviations: *—Results are presented as means ± standard deviations for parametric distribution, or medians and min–max for nonparametric distribution (verified using Shapiro–Wilk test *p* ≤ 0.05); **—Differences in parameters of groups WN and WH were assessed using Student’s *t*-test for parametric distribution and Mann–Whitney U test for nonparametric distribution; BMI—Body Mass Index; iAs—inorganic arsenic; WN—the group of workers with urinary tAs concentration within the norm; WH—the group of workers with urinary tAs concentration above the norm.

**Table 2:** Between-group comparison of the urinary tAs and As metabolite concentrations.

Parameter *	Both Group	WN	WH	*p* Value **
	*n* = 116	*n* = 75	*n* = 41	
tAs (µg/L)	27.3 (1.5–498.1)	20.6 (1.5–33.9)	54.7 (35.9–498.1)	0.0000
iAs (µg/L)	-	-	39.2 (10.0–87.8)	-
AsB (µg/L)	-	-	20.2 (2.5–433.0)	-
tAs (µg/g creat.)	19.0 (3.3–203.7)	14.6 (3.3–60.1)	30.4 (8.0–203.7)	0.0000
iAs (µg/g creat.)	-	-	20.1 (6.5–55.8)	-
AsB (µg/g creat.)	-	-	8.5 (0.8–170.8)	-

Abbreviations: *—Results are presented as medians and min–max for nonparametric distribution (verified using Shapiro–Wilk test *p* ≤ 0.05); **—Differences in parameters of groups WN and WH were assessed using Student’s *t*-test for parametric distribution and Mann–Whitney U test for nonparametric distribution; AsB—arsenobetaine; creat.—creatinine; iAs—inorganic arsenic; tAs—total arsenic; WN—the group of workers with urinary tAs concentration within the norm; WH—the group of workers with urinary tAs concentration above the norm.

**Table 3:** Dietary intake of selected nutrients involved in iAs metabolism.

Dietary Intake *	Both Groups	WN	WH	*p* Value **
	*n* = 116	*n* = 75	*n* = 41	
Methionine (mg/kg bm)	25.05 (8.43–70.48)	25.23 (8.43–70.48)	23.75 (14.69–54.47)	0.5220
Vitamin B_2_ (mg/kg bm)	0.02 (0.00–0.04)	0.02 (0.00–0.04)	0.02 ± 0.01	0.3554
Vitamin B_6_ (mg/kg bm)	0.02 (0.01–0.06)	0.02 (0.01–0.06)	0.02 ± 0.01	0.1506
Vitamin B_12_ (µg/kg bm)	0.04 (0.01–0.20)	0.03 (0.01–0.20)	0.04 ± 0.01	0.8515
Folate (µg/kg bm)	2.94 (1.03–8.98)	3.04 (1.13–8.98)	2.78 ± 0.80	0.1819
Zinc (mg/kg bm)	0.12 (0.03–0.30)	0.12 (0.03–0.30)	0.12 ± 0.04	0.5418

Abbreviations: *—Results are presented as means ± standard deviations for parametric distribution or medians and min–max for nonparametric distribution (verified using Shapiro–Wilk test *p* ≤ 0.05); **—Differences in parameters of groups WN and WH were assessed using Student’s *t*-test for parametric distribution and Mann–Whitney U test for nonparametric distribution; bm—body mass; WN—the group of workers with urinary tAs concentration within the norm; WH—the group of workers with urinary tAs concentration above the norm.

**Table 4:** Putatively annotated metabolites belonging to significantly changed pathways in analyses of differences between WN and WH.

Annotated Compounds’ Names (ID in HMDB)	*p* Value (WN vs. WH)	Pathway Name	Sub-Pathway Name
gamma-glutamylcysteine (HMDB0001049)	0.0000 *	Amino acid metabolism	Aspartate and asparagine metabolism
pyroglutamic acid (HMDB0000267)	0.0000 **		
D-2-hydroxyglutaric acid (HMDB0000606)	0.0000 *		
4-acetamidobutanoic acid (HMDB0003681)	0.0000 *		
urocanic acid (HMDB0000301)	0.0000 *		Histidine metabolism
3-mercaptolactic acid (HMDB0002127)	0.0034 *		Methionine and cysteine metabolism
L-cystine (HMDB0000192)	0.0000 *		
succinic acid (HMDB0000254)	0.0020 *	Carbohydrate metabolism	Butanoate metabolism
D-glucose (HMDB0000122)	0.0286 **		Glycolysis and Gluconeogenesis
D-xylulose (HMDB0001644)	0.0000 *		Pentose and Glucuronate Interconversions
2-ketobutyric acid (HMDB0000005)	0.0000 *		Propanoate metabolism
hydroxypropionic acid (HMDB0000700)	0.0000 **		
iduronic acid (HMDB0002704)	0.0000 **	Glycan biosynthesis and metabolism	Heparan sulfate degradation
N-acetylneuraminic acid (HMDB0000230)	0.0000 *		Keratan sulfate degradation
riboflavin (HMDB0000244)	0.0000 **	Metabolism of vitamins	Vitamin B2 metabolism
pyridoxine (HMDB0000239)	0.0000 *		Vitamin B6 metabolism
thymine (HMDB0000262)	0.0000 *	Nucleotide metabolism	Pyrimidine metabolism
uridine (HMDB0000296)	0.0000 **		
cytosine (HMDB0000630)	0.0000 *		
cytidine (HMDB0000089)	0.0000 *		
adenosine monophosphate (HMDB0000045)	0.0000 *	Many pathways	Aspartate and asparagine metabolism, Histidine metabolism, Methionine and cysteine metabolism, Butanoate metabolism, Glycolysis and Gluconeogenesis, Propanoate metabolism, Vitamin B2 metabolism, Pyrimidine metabolism
L-glutamic acid (HMDB0000148)	0.0000 *		Aspartate and asparagine metabolism, Histidine metabolism, Butanoate metabolism, Vitamin B9 metabolism
2-hydroxybutyric acid (HMDB0000008)	0.0000 *		Butanoate metabolism, Propanoate metabolism
D-glucuronic acid (HMDB0000127)	0.0000 *		Pentose and Glucuronate Interconversions, Heparan sulfate degradation, Hyaluronan metabolism
N-acetyl-D-glucosamine (HMDB0000215)	0.0000 **		Heparan sulfate degradation, Keratan sulfate degradation, Hyaluronan metabolism

Abbreviations: *—Student’s *t*-test; **—Mann–Whitney U test; HMDB—Human Metabolome Database; WN—the group of workers with urinary tAs concentration within the norm; WH—the group of workers with urinary tAs concentration above the norm.

**Table 5:** Relationship between the intake of dietary nutrients involved in iAs metabolism and putatively annotated metabolites belonging to significantly changed pathways in analyses of differences between WN and WH.

Correlation between Nutrient Intake *** and Metabolite	Both Group		WN		WH	
	R	*p*	R	*p*	R	*p*
vitamin B2 with cytosine	−0.1886 *	0.0464	−0.2554	0.0316	NS	
vitamin B6 with cytosine	−0.2175 *	0.0213	−0.2346	0.0489	NS	
vitamin B2 with D-glucuronic acid	NS		NS		−0.3956 *	0.0100
vitamin B6 with D-glucuronic acid	NS		NS		−0.3646 *	0.0190
vitamin B12 with D-glucuronic acid	NS		NS		−0.3479 *	0.0260
folate with D-glucuronic acid	−0.1996 *	0.0349	−0.2603*	0.0283	NS	
methionine with hydroxypropionic acid	−0.2163 *	0.0220	NS		NS	
vitamin B12 with hydroxypropionic acid	−0.2017 *	0.0330	NS		NS	
vitamin B2 with L-glutamic acid	NS		NS		−0.4211 **	0.0061
vitamin B6 with L-glutamic acid	NS		NS		−0.3653 **	0.0188
folate with L-glutamic acid	NS		NS		−0.3794 **	0.0144
methionine with N-acetyl-D-glucosamine	−0.1954 *	0.0390	−0.2728 *	0.0214	NS	
vitamin B2 with N-acetyl-D-glucosamine	−0.2504 *	0.0077	−0.3150 *	0.0075	−0.3253 *	0.0380
vitamin B6 with N-acetyl-D-glucosamine	−0.2374 *	0.0117	NS		NS	
vitamin B12 with N-acetyl-D-glucosamine	−0.1871 *	0.0482	−0.3261 *	0.0055	NS	
zinc with N-acetyl-D-glucosamine	−0.1917 *	0.0429	−0.2710 *	0.0223	NS	
vitamin B2 with N-acetylneuraminic acid	−0.1915 *	0.0431	NS		NS	
vitamin B6 with N-acetylneuraminic acid	−0.2246 *	0.0173	NS		NS	
vitamin B12 with N-acetylneuraminic acid	NS		NS		−0.3171 *	0.0430
vitamin B2 with pyroglutamic acid	NS		−0.2832 *	0.0167	NS	
vitamin B6 with pyroglutamic acid	NS		−0.2430 *	0.0412	NS	
vitamin B12 with pyroglutamic acid	NS		−0.2667 *	0.0246	NS	
folate with pyroglutamic acid	−0.1894 *	0.0454	NS		NS	
zinc with pyroglutamic acid	NS		−0.2548 *	0.0320	NS	
vitamin B2 with uridine	−0.1944 *	0.0400	NS		NS	
vitamin B6 with uridine	−0.1955 *	0.0388	NS		NS	
vitamin B12 with uridine	NS		−0.2559 *	0.0313	−0.3308 *	0.0350
methionine with urocanic acid	NS		−0.2569 *	0.0305	NS	
vitamin B6 with urocanic acid	NS		−0.2457 *	0.0389	NS	
folate with urocanic acid	−0.2067 *	0.0288	−0.2665 *	0.0247	NS	
zinc with urocanic acid	NS		−0.2722 *	0.0217	NS	
folate with 3-mercaptolactic acid	NS		NS		0.3337 *	0.0330
zinc with 3-mercaptolactic acid	NS		NS		0.3658 *	0.0190
methionine with succinic acid	NS		NS		0.3284 **	0.0361
folate with succinic acid	NS		NS		0.3359 *	0.0320
zinc with succinic acid	NS		NS		0.3498 *	0.0250

Abbreviations: *—Pearson correlation coefficient; **—Spearman rank correlation coefficient; ***—intake of nutrients calculated per kg of body weight; NS–not statistically significant; R—correlation coefficient; *p*—*p* value; WN—the group of workers with urinary tAs concentration within the norm; WH—the group of workers with urinary tAs concentration above the norm.

## Data Availability

The data presented in this study are available in article and supplementary materials.

## Supplementary Materials

The following supporting information can be downloaded at: https://www.mdpi.com/article/10.3390/metabo13010070/s1, Table S1: Dietary intake of selected nutrients involved in iAs metabolism regarding to the EAR norm.
